# Severe and uncomplicated falciparum malaria in children from three regions and three ethnic groups in Cameroon: prospective study

**DOI:** 10.1186/1475-2875-11-215

**Published:** 2012-06-24

**Authors:** Eric A Achidi, Tobias O Apinjoh, Judith K Anchang-Kimbi, Regina N Mugri, Andre N Ngwai, Clarisse N Yafi

**Affiliations:** 1 Department of Medical Laboratory Science, Faculty of Health Sciences, University of Buea, PO Box 63, Buea, Cameroon; 2 Department of Biochemistry and Molecular Biology, Faculty of Science, University of Buea, Buea, Cameroon; 3 Department of Zoology and Animal Physiology, University of Buea, Buea, Cameroon; 4 Department of Biology and Animal Physiology, University of Yaoundé I, Yaoundé, Cameroon

**Keywords:** Severe malaria, Uncomplicated malaria, Children, Location, Ethnicity

## Abstract

**Background:**

To identify the factors that account for differences in clinical outcomes of malaria as well as its relationship with ethnicity, transmission intensity and parasite density.

**Methods:**

A prospective study was conducted in nine health facilities in the Centre, Littoral and South West regions of Cameroon, and in three ethnic groups; the Bantu, Semi-Bantu and Foulbe. Children aged one month to 13 years, with diagnosis suggestive of malaria, were recruited and characterized using the WHO definition for severe and uncomplicated malaria. Malaria parasitaemia was determined by light microscopy, haematological analysis using an automated haematology analyser and glucose level by colorimetric technique.

**Results:**

Of the febrile children screened, 971 of the febrile children screened fulfilled the inclusion criteria for specific malaria clinical phenotypes. Forty-nine (9.2%) children had cerebral malaria, a feature that was similar across age groups, ethnicity and gender but lower (*P* < 0.004) in proportion in the Centre (3.1%, 5/163) compared to the Littoral (11.3%, 32/284) and South West (13.6%, 12/88) regions. Severe anaemia was the most frequent severe disease manifestation, 28.0% (248/885), which was similar in proportion across the three ethnic groups but was more prevalent in females, less than 60 months old, and the Centre region. About 20% (53/267) of the participants presented with respiratory distress, a clinical phenotype independent of age, gender and ethnicity, but highest (*P* < 0.001) in the Centre (55%, 11/20) compared to the Littoral (27.3%, 3/11) and South West (16.5%, 39/236) regions. Uncomplicated malaria constituted 27.7% (255/920) of hospital admissions and was similar in proportion with gender and across the three ethnic groups but more prevalent in older children (≥ 60 months) as well as in the South West region. The density of malaria parasitaemia was generally similar across clinical groups, gender and ethnicity. However, younger children and residents of the Centre region carried significantly higher parasite loads, with the burden heavier in the Semi-Bantu compared to their Bantu (*P* = 0.009) and Foulbe (*P* = 0.026) counterparts in the Centre region. The overall study case fatality was 4.8 (47/755), with cerebral malaria being the only significant risk factor associated with death. Severe anaemia, though a common and major clinical presentation, was not significantly associated with risk of death.

**Conclusion:**

About half of the acutely febrile children presented with severe malaria, the majority being cases of severe malaria anaemia, followed by respiratory distress and cerebral malaria. The latter two were less prevalent in the Centre region compared to the other regions. Cerebral malaria and hyperpyrexia were the only significant risk factors associated with death.

## Background

Malaria remains one of the most widespread infectious diseases of humankind, threatening approximately half the world’s population and causing debilitating illness in more than 216 million people. Morbidity and mortality is particularly high in sub-Saharan Africa, with children below five years at the greatest risk [1]. *Plasmodium falciparum*, the deadliest of the malaria parasite species, kills a child every 30 seconds in Africa [2] and also wreaks significant economic havoc in highly endemic areas, substantially decreasing Gross Domestic Product (GDP) of affected countries relative to malaria-free regions [3].

In endemic areas, malaria accounts for 25–35% of all outpatients’ visits, 20–45% of hospital admissions and 15–35% of hospital deaths [4]. The clinical spectrum of paediatric *P. falciparum* infections range from asymptomatic parasite carriage to a febrile disease that may develop into severe, life-threatening illness [5]. Mortality from malaria is associated largely with the parasite’s ability to induce severe complications, presenting as severe anaemia, cerebral malaria and metabolic acidosis, manifested clinically as respiratory distress. Other severe malaria manifestations at enrolment include multiple or prolonged convulsions, hyperlactataemia, hyperparasitaemia, hypoglycaemia, hyperpyrexia and intravascular haemolysis [6,7].

The factors that determine malaria severity are not completely understood. Despite the scaling up of the provision of insecticide-treated nets and the increasing use of the most rapidly parasiticidal artemisinin derivatives [8,9], the risk of and mortality from malaria still remain significantly high [1,2]. Studies on factors associated with increased risk of developing severe malaria and death, may provide additional understanding of the course of severe malaria, and, eventually, lead to improved case management, and the development of drugs and vaccines for malaria.

Studies on paediatric malaria in Cameroon are limited [10] and although several studies, at various settings in Africa, have attempted to delineate the epidemiology of clinical malaria, the data have shown significant variability across various transmission zones [6,7,11-14]. Nevertheless, severe malaria features may change according to a number of factors including the genetic characteristics of the population, malaria epidemiology, health-seeking behaviour, non-malaria co-morbidity, clinical assessment and local case management [14]. There is, therefore, a need for more site-specific data in order to appreciate the complete clinical and epidemiological picture needed for efficient testing of candidate malaria vaccines and other control tools in different endemic sites. Furthermore, how the peripheral parasite density varies with transmission or influences the different types of manifestations of specific clinical features is poorly described [13].

A hospital-based study was, therefore, undertaken to determine the factors that account for different clinical outcomes of malaria as well as its relationship with ethnicity, transmission intensity and parasite density in young children from three regions with distinct ecological conditions across Cameroon. The prevalence of the clinical phenotypes in hospitals was used as a proxy measure, although malaria disease patterns related to transmission are best studied using incidence data [15].

## Methods

### Study area

A prospective study was conducted in four towns distributed in three regions of Cameroon, namely: Yaounde in the Centre; Douala in the Littoral; and Buea and Limbe in the South West. The study sites included hospitals (Bota District Hospital - Limbe, Laquintinie Hospital - Douala, Mother and Child Hospital - Yaounde, Regional Hospital - Limbe and Regional Hospital Annex - Buea) and health centres (Bokova Health Centre, Mount Mary Health Centre - Buea and PMI Down Beach - Limbe). Except for Mount Mary, the chosen health facilities were the main government institutions in the selected towns, also receiving patients from surrounding areas. Although malaria is endemic throughout Cameroon, the country has very different geographical and epidemiologic strata that may alter the course of the infection [16].

The central region (Yaoundé) is located within the rainforest belt of central Africa [17] and has the Guinea-type equatorial climate [16]. This is characterised by fairly constant temperatures ranging from 17°C to 30°C (mean = 23.1°C) [18], abundant rainfall (1,500–2,000 mm), with the average relative humidity index ranging from 85% to 90%, and four distinct seasons: two rainy seasons (March–May/June, September–November) and two dry seasons (December–February, June/July–August). Maximal transmission of malaria occurs during and immediately following the two rainy seasons [16-18]. Only *Anopheles gambiae* and *Anopheles funestus* contribute to malaria transmission in urban Yaoundé and their distribution is seasonal. The entomological inoculation rate has been estimated at 34 infectious bites per person per year [19]. The Mother and Child Hospital is a referral hospital for children and mothers, located in the heart of the city of Yaoundé, it also attracts patients from neighbouring villages, such as Simbok and Etoa that are stable, rural, farming communities with fields irrigated by water from the Mefou and Biyeme Rivers. Inhabitants of this region are of the Ewondo tribe and part of the Bantu ethnic group.

The South Western and Littoral regions have a Cameroonian-type equatorial climate characterized by fairly constant temperatures and two seasons: a short dry season (November–March) and a long rainy season (March-November) with abundant precipitation (2,000–10,000 mm) [16]. In the Mt Cameroon region of the south-west, the mean annual rainfall is 2,625 mm, relative humidity is constantly high (75%–80%), and the temperature varies from 18°C in August to 35°C in March [20]. In general, malaria transmission is intense and perennial in the Littoral, and South Western regions, with peak periods corresponding to the rainy seasons [16].

In the Mount Cameroon region of the South West, human malaria is meso-endemic during the dry season but becomes hyper-endemic in the rainy season, with incidence peaking in July–October. The prevalence of malaria parasitaemia in the low-altitude areas ranges from 30% in the dry season to 84% in the rainy season [21,22]. *Anopheles gambiae* is the dominant (most aggressive and most active [20,23,24] of the three malaria vectors (*An. gambiaeAn. funestus* and *Anopheles nili*), accounting for up to 72.7% of transmission [23]. Infection rates by *An. gambiae* are as high as 287 infective bites/person/year [23] and overall EIR estimated recently at 3.93 infective bites/person/night [24]. *Plasmodium falciparum* accounts for up to 96% of malaria infections in this area [23].

### Study design and population

The study conducted between May 2003 to December 2005 and April 2007 to August 2008 involved children (aged one month to 13 years) with acute/mild illness, reporting for consultation at the emergency/outpatient units of nine health facilities in the Centre, Littoral and South West regions. The criteria for diagnosis and enrolment included the standard WHO definitions [25] i.e. the presence of asexual parasitaemia and at least one of the following: cerebral malaria (impaired consciousness or unarousable coma [(Blantyre coma score ≤ 2, corrected for hypoglycaemia (blood glucose < 2.2 mmol/l)] and no record of recent severe head trauma, neurological disease or any other cause of coma); severe malaria anaemia (haemoglobin < 5g/dL or haematocrit < 15%), no cases of severe bleeding); convulsions before/during admission, respiratory distress (presence of alar flaring, intercostals or subcostal chest recession, use of accessory muscles of respiration, or abnormally deep respiration); hypoglycaemia (blood glucose <2.2mmol/L); hyperpyrexia (axillary temperature ≥40^0^C); hyperparasitaemia (>250,000 parasite/μL) as well as uncomplicated malaria (fully conscious with haemoglobin ≥ 8g/dL and no signs of severity and/or evidence of vital organ dysfunction).

**Figure 1:**
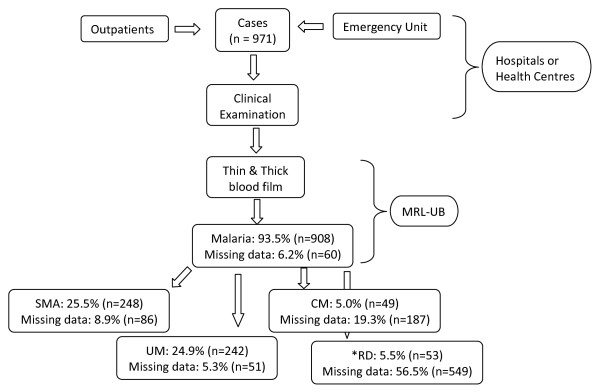
Schematic process of the screening and enrolment of febrile children in nine health institutions in the Centre, Littoral and South West regions of Cameroon.

Individuals who fulfilled the specific inclusion criteria and volunteered to participate after adequate sensitization on the project objectives, methods and possible benefits/risks were enrolled into the study (Figure 1). Caregivers were interviewed about the presenting symptoms and study physicians documented findings of clinical examination including weight and vital signs. Participants were screened and assigned to their clinical disease categories before any treatment was administered. In addition, the course of illness, including vital signs, haematological parameters and peripheral blood parasitaemia, were documented for each study child from the time of enrolment to the time of exit from the hospital. Patients were seen twice daily while on admission and vital signs measured daily. However, peripheral blood parasitaemia and haematological parameters were measured at enrolment and on discharge from the hospital, except otherwise requested by study clinicians.

### Exclusion criteria

Patients with a history of anti-malarial treatment within two weeks prior to admission or were sickling positive were excluded. Patients were also excluded from the study based on evidence of other infectious disease, such as typhoid, gastroenteritis, meningitis, malnutrition or upper respiratory tract infections or any other identified cause of anaemia than malaria.

### Clinical evaluation and management

Medical doctors and/or nurses were involved in case management at the different health facilities. The participants’ axillary temperature was taken using a digital thermometer except for infants (<2 years) for whom rectal measurements were preferred. Information relating to age, gender, area of residence, mid-upper-arm circumference, clinical observations (spleen size, etc) and management/outcome were documented using a standard Case Report Form (CRF).

Children were treated using a uniform protocol based on standard recommendations [9,26] consisting of parenteral quinine at 20 mg/kg loading dose followed by 10 mg/kg maintenance dose every 12 hours typically for three days or until the patient was able to swallow, at which point he/she was switched to an oral dose of 10 mg/kg eight-hourly to complete a seven-day course of treatment. Uncomplicated malaria cases were mainly first treated with artemisinin-combination therapy (mainly artesumate - amodiaquine) according to national guidelines and quinine sulphate was used as the rescue drug. In addition to the specific treatment for *P. falciparum* infection, children received supportive therapy in terms of haemotransfusion for severe anaemia, intravenous glucose for hypoglycaemia and intravenous fluid for severe dehydration. Paracetamol suppositories were administered for pyrexia, diazepam for seizures, and nasal oxygen for respiratory distress according to national guidelines.

### Laboratory procedures

At enrolment, peripheral malaria blood smears, full blood count and blood glucose were done for all participants. Thick and thin blood films were made, thin film fixed with methanol and both thin and thick film stained with 5% Giemsa (Sigma, St. Louis, USA). The malaria parasitaemia and density were determined in the thick blood smear under oil immersion with the 100x objective, 10x eyepiece of a binocular Olympus microscope (Olympus Optical Co., Ltd, Japan). A smear was only considered negative if no malaria parasites were seen in 50 high power fields (HPF). With each positive smear, the level of parasitaemia was estimated by counting the parasites against at least 200 leucocytes and using the corresponding leucocyte count to calculate the number of parasites/μl blood [27]. The malaria parasite species were detected in the thin blood film. Whole blood specimens were analysed for full blood count using automated Teco diagnostcs haematology analyser; blood glucose levels were determined using colorimetric techniques (Chronolab AG, Switzerland).

### Administrative approvals and consent

Ethical clearance was obtained from the Institutional Review Board of the University of Buea and South West Regional Delegation of Public Health. A health facility was only included in the study with the approval of its Director and participants were only enrolled if their caregivers gave written informed assent. The consenting process involved the explanation of the content of the information sheets to caregivers by a responsible research team member in the language he/she best understood and opportunities were given for questions/clarification. Emphasises was laid on the voluntary nature of participation and the fact that refusal had no influence on the quality of medical care from the health facility.

### Data and statistical analysis

All study data were captured on a structured case report form bearing subject demographic and identification numbers. All forms were reviewed before being double entered onto a computer. Data missing at random was dealt with by Partial case-wise deletion. Statistical analyses were performed using SPSS Statistics 17.0 (SPSS Inc., Chicago, USA). Levels of parasitaemia were log-transformed before analysis. The significance of differences in prevalence were explored using Pearson’s Chi-square or Fisher’s Exact test whereas the differences in group means were assessed using Student’s t tests, analyses of variance (ANOVA) with Post Hoc Tukey test or Kruskal-Wallis test. Multivariate analysis was undertaken using linear or logistic regression and included the covariates: age and clinical malaria phenotype. A difference giving a P-value ≤ 0.05 was considered statistically significant.

## Results

### Baseline characteristics

**Figure 2:**
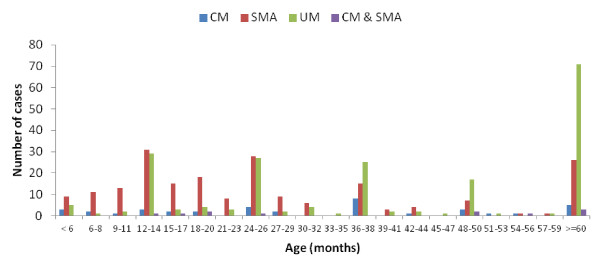
Age-specific distribution of severe and uncomplicated malaria in three regions of Cameroon.

**Figure 3:**
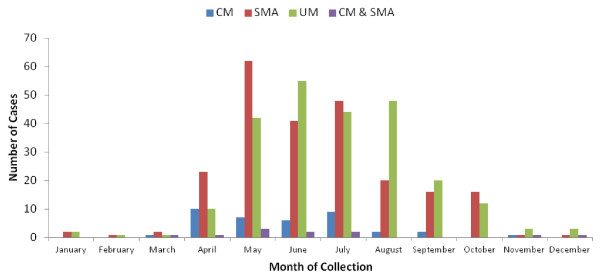
Seasonal prevalence of severe and uncomplicated malaria in three regions of Cameroon.

A total of 971 children satisfied the criteria for specific malaria clinical phenotypes and were enrolled into the study. These participants were admitted in the Centre (164, 16.9%), Littoral (292, 30.1%) and South West (515, 53%) regions, from the Bantu (385, 39.6%), Semi-Bantu (416, 42.8%), Foulbe (31, 3.2%) and other (139, 14.3%) ethnic groups. The ethnicity was defined as the self-reported ethnic group of the mother (or father if the mother’s could not be obtained). About 53.3% (516/969) of the total study participants were males and majority (57.7%, 492/852) aged below 36 months (Figure 2) recruited during the rainy season months (95.8%, 918/957) (Figure 3).

The median age, weight and mid upper arm circumference were respectively 25.88 months (range: 1–156), 11.00 kg (range: 3–53) and 15.00 cm (range: 5–27). At baseline, the mean temperature, haemoglobin and geometric mean parasite density were 38.6°C (range: 35.7-43.0), 6.2 g/dl (range 1.3-14.7) and 8,873 parasites/μL respectively. The proportion with fever (axillary temperature ≥ 37.5°C) and anaemia (haemoglobin < 11 g/dl) were 87.9% (678/771) and 94.7% (889/939) respectively. Other enrolment symptoms were capillary refill less than two seconds (61%), inability to sit unaided or breastfeed (33.8%), dehydration (8.4%), fitting (7.7%), jaundice (5.3%), bipedal oedema (3.4%) and neck stiffness or bulging fontanelle (2.6%). Reported fever, convulsions, malarial chemotherapy and anti-convulsants use prior to admissions were 98.1% (773/788), 32.3% (246/761), 74.7% (343/459) and 21.0% (97/462) respectively.

### Major clinical and prognostic features

#### Cerebral malaria

Although 71 (13.2%) of the children were comatose (Blantyre coma score ≤2), only 6.3% (49/784) had cerebral malaria (CM) (Blantyre coma score ≤ 2 and plasma glucose >2.2 mmol/l). The mean age (± SD) of CM patients was 37.85 ± 30.15 months. The frequency of CM in children less than 60 months old (9.3%) was similar to those 60 months and above (8.8%) as well as in females (10.0%, 26/259) compared to males (8.3%, 23/276). The proportion of children with cerebral malaria was lower (P < 0.004) in the Centre (3.1%, 5/163) compared to the Littoral (11.3%, 32/284) and South West (13.6%, 12/88) regions but similar in the Bantu (9.7%, 21/217), Semi-Bantu (8.3%, 23/277) and Foulbe (8.0%, 2/25) ethnic groups.

Fewer children with cerebral malaria had severe anaemia (22.4%, 11/49) or respiratory distress (28.6%, 4/14) compared to those without cerebral malaria [(28.8%, 134/466) and (33.3%, 28/83)] respectively (Figure 3). However, cerebral malaria patients tended to be more hyperparasitaemic (12.5%, 6/48) and hyperpyrexic (19.1%, 9/47) compared to their non-cerebral malaria counterparts (6.0%, 28/463) and (13.4%, 64/477) respectively. Apart from the higher (P = 0.019) mean haemoglobin level in cerebral malaria patients (6.29 ± 2.34) g/dl compared to that of children without (5.58 ± 1.98), mean age (37.85 ± 30.15) months, plasma glucose (6.41 ± 3.57) mmol/l and geometric mean parasite density (8,578 parasites/μL) were similar to those of non cerebral malaria children. The total case fatality among children with cerebral malaria (23.4%, 11/47) was higher than that of children without cerebral malaria (5.6%, 26/467).

#### Severe malaria anaemia

**Figure 4:**
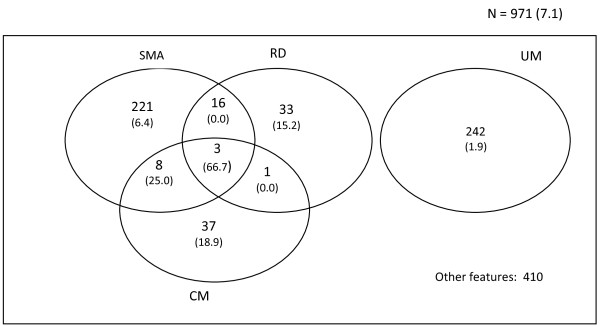
Venn diagram of proportions, overlap and mortality of major clinical subgroups of malaria.

Severe malaria anaemia (SMA) was the most frequent severe manifestation of malaria, 28.0% (248/885) and was similar in proportion across the three ethnic groups but more prevalent in females, less than 60 month of age and the Centre region (Table 1). The mean age (± SD) of SMA patients was 30.96 ± 28.12 months. More children with severe malaria anaemia (P = 0.007) had respiratory distress (30.6%, 19/62) compared to those without (14.9%, 26/174) (Figure 4) while fewer severe malaria anaemic children (P = 0.002) were hyperparasitaemic (2.5%, 6/242) compared to their non severe malaria anaemic counterparts (8.4%, 53/640). The overlap of severe anaemia with cerebral malaria and hyperpyrexia were 7.6% (11/145) and 12.4% (25/202) respectively (Figure 4, Table 2). More children (P < 0.001) with severe malaria anaemia at enrolment 86% (172/200) received blood transfusions compared to cases without severe malaria anaemia 58.3% (299/513).

**Table 1:** Variation in the major clinical disease phenotypes with age, gender, ethnicity and location of residence

**Parameters**	**N**	**CM**	**P value**	**N**	**SMA**	**P value**	**N**	**UM**	**P value**	**N**	**RD**	**P value**
Age group (months)												
< 60	562	41 (7.3)	0.420	637	187 (29.4)	0.009	662	130 (19.6)	<0.001	284	44 (15.5)	0.143
≥ 60	148	8 (5.4)		158	30 (19.0)		167	71 (42.5)		95	9 (9.5)	
Gender												
Male	409	23 (5.6)	0.449	469	118 (25.2)	0.042	487	133 (27.3)	0.473	192	23 (15.6)	0.082
Female	375	26 (6.9)		415	130 (31.3)		432	109 (25.2)		230	30 (10.0)	
Ethnicity												
Bantu	313	21 (6.7)	0.996	358	98 (27.4)	0.368	368	103 (28.0)	0.031	177	26 (14.7)	0.827
Semi-Bantu	352	23 (6.5)		388	111 (28.6)		409	84 (20.5)		169	21 (12.4)	
Foulbe	30	2 (6.7)		30	5 (16.7)		31	5 (16.1)		7	1 (14.3)	
Location												
Centre	163	5 (3.1)	<0.001	162	61 (37.7)	0.004	163	20 (12.3)	<0.001	39	11 (28.2)	0.008
Littoral	285	32 (11.2)		277	63 (22.7)		288	19 (6.6)		29	3 (10.3)	
South West	336	12 (3.6)		446	124 (27.8)		469	203 (43.3)		354	39 (11.0)	
Transmission												
Seasonal	163	5 (3.1)	0.059	162	61 (37.7)	0.003	163	20 (12.3)	<0.001	39	11 (28.2)	0.002
Perennial	621	44 (7.1)		723	187 (25.9)		757	222 (29.3)		383	42 (11)	

Each participant has been categorised into one group only which clinicians felt was the primary phenotype; Samples numbers in some of the clinical groups are lower because some parameter estimates were not taken.

**Table 2:** Characteristics of the major clinical phenotypes

**Parameter**	**N**	**CM**	**N**	**SMA**	**N**	**UM**	**N**	**RD**	**P-value**
Hyperpyrexia, n (%)	47	9 (19.1)	191	24 (12.6)	154	29 (18.8)	32	9 (28.1)	0.110
Hyperparasitaemia, n (%)	48	6 (12.5)	231	5 (2.2)	228	14 (6.1)	26	3 (11.5)	0.008
Hypoglycaemia, n (%)	49	-	187	24 (12.8)	177	18 (10.2)	26	4 (15.4)	0.617
Fatality, n (%)	47	11 (23.4)	187	11 (5.4)	154	3 (1.9)	33	5 (15.2)	<0.001
Mean age ± SD (months)	49	37.85 ± 30.15	205	29.96 ± 27.33	201	51.41 ± 38.81^♣^	33	40.28 ± 34.33	<0.001
Mean haemoglobin ± SD (g/dl)	49	6.29 ± 2.34	225	3.65 ± 0.83	222	9.60 ± 1.31	31	7.71 ± 2.27	<0.001
*GMPD, parasites/μL	48	8,578	231	7,754	228	6,791	26	17,512	0.346
Mean spleen size ± SD (cm)	41	0.95 ± 1.66	124	1.51 ± 2.37	104	1.08 ± 1.94	15	3.13 ± 3.04^§^	0.004
Mean plasma glucose ± SD (mmol/l)	49	6.41 ± 3.57	187	5.91 ± 4.41	177	6.23 ± 6.33	26	4.63 ± 4.25	0.474

^♣^Age significantly higher than the corresponding values for SMA (P < 0.001) and CM (*P* = 0.050).

^§^Spleen size significantly higher than the corresponding values for CM (*P* = 0.005), SMA (*P* = 0.033) and UM (*P* = 0.004).

The haemoglobin values are significantly different between all four clinical groups; each participant has been categorised into one group only which clinicians felt was the primary phenotype. Samples numbers in some of the clinical groups are lower because some parameter estimates were not taken.

The total case fatality among children with severe anaemia (7.6%, 15/198) was similar to that of children without SMA (5.5%, 28/509) (Table 2).

#### Respiratory distress

About 20% (53/267) of participants assessed presented with respiratory distress (RD). The proportion of children with this phenotype was independent of age, gender and ethnicity but highest (P < 0.001) in the Centre (55%, 11/20) compared to the Littoral (27.3%, 3/11) and South West (16.5%, 39/236) regions. The geometric mean parasite load of these participants was also similar to that of patients without respiratory distress (Table 2). Respiratory distress was significantly higher (P = 0.007) in severe anaemia (30.6%, 19/62) patients compared to those without SMA (14.9%, 26/174). However, the proportion of children with respiratory distress was similar in cerebral malaria patients (28.8%, 4/14) and hyperparasitaemics (21.1%, 4/19) compared to those without CM (33.7%, 28/83) and hyperparasitaemia (18.7%, 41/219 respectively). The case fatality of respiratory distress was 13.5% (7/52) and was significantly increased to 66.7% (2/3) when the respiratory distress overlapped with cerebral malaria plus severe anaemia (Figure 4).

#### Uncomplicated malaria

Uncomplicated malaria (UM) was also a very frequent manifestation constituting 26.3% (242/920) of hospital admissions. This clinical phenotype was similar in proportion with gender and across the three ethnic groups but more prevalent in older children as well as in the South West region (Table 2). There were fewer uncomplicated malaria children with respiratory distress (13%, 13/100) (Figure 4) and hyperpyrexia while the proportion of hyperparasitaemia and hypoglycaemia was similar to those of patients without the phenotype (Table 2). The total case fatality among children with uncomplicated malaria (3.0%, 5/167) was significantly increased to 15.4% (2/13) when it overlapped with respiratory distress (Figure 4).

### Parasitaemia

**Figure 5:**
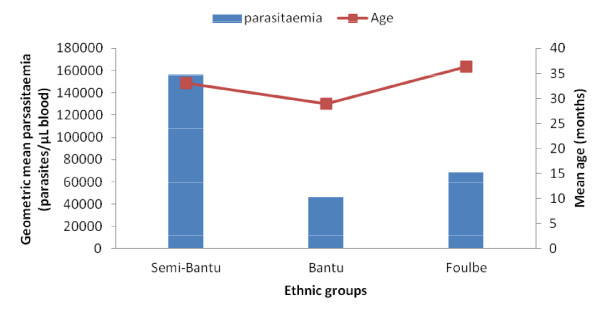
Variation of geometric mean malaria parasitaemia density and age with ethnicity of children from the central region of Cameroon.

The geometric mean density of parasitaemia was generally similar in cerebral malaria (8,578 parasites/μL), severe malaria anaemia (7,754 parasites/μL) and uncomplicated malaria (6,791parasites/μL) patients as well as with gender and ethnicity. Younger children and residents of the Centre region, however, carried significantly higher parasite loads (Table 3). Furthermore, in the Centre region, there was a significant variation in parasitaemia with ethnicity (*P* = 0.003), with higher densities in the Semi-Bantu compared to their Bantu (*P* = 0.009) and Foulbe (*P* = 0.026) counterparts (Figure 5). Nevertheless, age was similar across ethnicity in this region. Although the difference in parasitaemia with gender was insignificant in CM and SMA, females with uncomplicated malaria had higher (*P* = 0.007) parasite loads compared to their male counterparts.

**Table 3:** Variation in geometric mean malaria parasitaemia density with age, gender, ethnicity and location across the major clinical subgroups

**Parameters**	**N**	**All subjects**	**N**	**CM**	**n**	**SMA**	**n**	**UM**	**P value**
Age group									
< 60	642	10,451	40	8,940	177	10,089	120	6,791	0.415
≥ 60	159	5,998	8	6,978	27	2,037	68	8,954^♣^	0.046
*P* value		0.013		0.831		0.001		0.504	
Gender									
Male	472	7,773	22	7,968	110	7,636	126	4,405	0.223
Female	421	10,293	26	9,130	121	7,862	102	11,592	0.582
*P* value		0.087		0.876		0.835		0.007	
Ethnicity									
Bantu	360	9,134	21	6,246	90	7,962	96	7,638	0.927
Semi-Bantu	391	11,171	23	10,993	103	9,670	79	10,194	0.973
Foulbe	30	9,387	2	4,205	5	1,336	5	13,402	0.522
*P* value		0.539		0.789		0.156		0.738	
Location									
Centre	162	17,640^‡^	5	9,908	60	10,336	20	43,031^§^	0.014
Littoral	281	7,292	32	6,296	58	5,243	18	7,385	0.873
South West	452	7,836	11	19,756	113	8,136	190	5,540	0.161
*P* value		0.001		0.547		0.258		0.005	
Transmission									
Seasonal	162	17,640	5	9,908	60	10,336	20	43,031^§^	0.014
Perennial	733	7,623	43	8,435	171	7,010	208	5,686	0.555
*P* value		<0.001		0.910		0.296		0.001	

Each participant has been categorised into one group only which clinicians felt was the primary phenotype; ^♣^Significantly higher than the corresponding values for SMA (*P* = 0.035).

^§^Significantly higher than the corresponding values for SMA (*P* = 0.006) and patients from the South West region (*P* = 0.004).

^‡^Significantly higher than the corresponding values for the Littoral (P = 0.001) and South West regions (*P* = 0.002).

Each participant has been categorised into one group only which clinicians felt was the primary phenotype; Samples numbers in some of the clinical groups are lower because some parameter estimates were not taken.

### Factors associated with malaria death

The overall study case fatality was 4.8 (47/755). This was similar in males and females (6.6% *vs* 5.8%), Bantu, Semi-Bantu and Foulbe ethnic groups (5.7% *vs* 6.0% *vs* 6.9%) as well as the Centre, Littoral and South West regions (8.7% *vs* 6.7% *vs* 4.6%). Their baseline presentations were: age less than 60 months 91.1% (41/45), respiratory distress 46.7% (7/15), severe malaria anaemia 34.9% (15/43), cerebral malaria 29.7% (11/37), hyperpyrexia 27.3% (12/44), hypoglycaemia 21.4% (9/42) and hyperparasitaemia 11.1% (5/45). Bivariate analysis (Table 4) showed that cerebral malaria, hyperpyrexia, hypoglycaemia and respiratory distress were the risk factors associated with most deaths but logistic regression analysis showed that cerebral malaria and hyperpyrexia were associated with malaria death.

**Table 4:** Prognostic indicators at the time of enrolment

**Outcome**	**Prevalence [n (%)]**	**Fatalities [n (%)]**	**Univariate analysis**		**Multivariate analysis**	
			**χ**^**2**^	**P value**	**OR**	**95%**
						**CI**
Cerebral malaria	49 (5.0)	11 (22.4)	20.337	< 0.001	20.17	1.82-223.47
Severe malaria anaemia	248 (25.5)	15 (7.6)	1.101	0.294	2.65	0.38-18.56
Uncomplicated malaria	242 (24.9)	3 (1.9)	5.541	0.019	1.18	0.09-15.83
Hyperparasitaemia	58 (6.0)	5 (9.8)	1.119	0.290	0.14	0.01-1.70
Hyperpyrexia	116 (11.9)	12 (10.8)	5.479	0.019	11.33	1.60-80.02
Hypoglycaemia	89 (9.2)	9 (12.9)	4.601	0.032	0.96	0.87-1.06
Respiratory distress	53 (5.5)	7 (13.5)	7.134	0.013	3.22	0.36-29.02
Hypoglycaemia	89 (9.2)	9 (12.9)	4.601	0.032	0.96	0.87-1.06
Respiratory distress	53 (5.5)	7 (13.5)	7.134	0.013	3.22	0.36-29.02

N = 971.

## Discussion

Studies on severe malaria in Cameroonian children are limited, in spite of the several advantages that severe malaria offers over other potential endpoints (such as death) for future malaria vaccine efficacy trials, the scaling up of the provision of insecticide-treated nets and the adoption of new effective therapeutics. Furthermore, it is not clear how the peripheral parasite density varies with transmission or influences the different types of severe malaria clinical features. This study sets out to determine the factors that account for different clinical outcomes of malaria parasite infection as well as its relationship with ethnicity, transmission intensity and parasite density in young children from three regions with distinct ecological conditions across Cameroon. The results showed that significant hospital admissions and numbers of severe malaria cases exist in the Centre, Littoral and South West regions of Cameroon and that severe malaria in this setting may represent an adequate vaccine trial endpoint [7,28].

The current study showed that over a period of 48 months, 971 (49.4%) hospital-based malaria cases were recruited, the majority (57.7%, 492/852) aged below 36 months, with a significant decreasing trend towards increasing age (Figure 2). The proportion of severe cases (57.2%) was highest in children less than 24 months as reported elsewhere [7,14] supporting the observed association between age and disease severity. As in some settings, the predominant manifestation was severe malarial anaemia. Other clinical and laboratory manifestations were also consistent with what had earlier been reported in other endemic settings [6,7,11,13,14,29].

The mean age of children with severe malaria anaemia and cerebral malaria was 30.96 and 37.85 months respectively (Table 1), consistent with results from previous studies that severe anaemia is more common in infants and young children <36 months while cerebral malaria is commoner in older children [14,25]. Nevertheless, this should be interpreted with caution, as the assessment of neurological status in children younger than eight months, and therefore the percentage of those with low Blantyre coma scores, is unreliable in this age group [30].

There was a significant variation in the age of paediatric malaria admissions and the proportion of the different clinical groups in the three regions (Table 1). The latter may reflect differences in parasite and/or vector virulence or transmission intensities, but may also echo the difficulties in measuring cerebral malaria (and perhaps other phenotypes) in very young children [29]. The observed age difference is consistent with the view that the speed of acquired anti-disease immunity depends on the frequency of parasite exposure since birth. Such variation in background immunity may also explain the differences in the age of presentation of severe malaria as reported previously [6,14] or other undefined variables. More importantly the difference in peak age of hospitalized malaria presentation has implications for the design of prevention strategies planned for the control of malaria in Africa. The benefit of targeted intervention in the use of bed nets to the highest risk groups and intermittent preventive treatment in infants (IPTi) must adapt to include increasingly older age risk groups in order to have greater impact. Universal coverage is now the aim whenever resources allow.

It is not surprising that children aged below 60 months carried the greater malaria parasite burden compared to their older counterparts (Table 2) and that parasitaemia correlated inversely with age (data not shown). Residents of malaria-endemic areas have long periods of malaria parasitaemia punctuated by episodic clinical attacks that decrease in frequency with age. This pattern has been explained by the acquisition of anti-disease/anti-toxic immunity in early childhood and later anti-parasitic immunity following repeated exposure to malaria [31]. However, parasite loads were similar across clinical groups, except in the Centre region (Table 2), suggesting that the density of malaria infection is not a significant factor in determining disease severity. Nevertheless, the observed differences in the Centre corroborate previous reports [13,14] suggesting that heavy *P. falciparum* malaria parasitaemia may be important in the development of seizures, severe anaemia and impaired consciousness or deep coma. Additionally, sequestered parasites have been implicated in the pathology of severe malaria manifestations [32] so that peripheral parasitaemia, on average, is not expected to show significant associations with clinical manifestations. Taken together, correlation between severity of disease and peripheral parasitaemia is not clear-cut and the latter may only play a small role in the overall complexity of severe malaria syndromes.

Surprisingly, malaria parasitaemia varied with ethnicity in the Centre region (Figure 5). This is unlikely due to age since age was similar (*P* = 0.403) across these ethnic groups in the region. Also, since these individuals live in the same region, location cannot account for the difference observed. The finding seems to suggest that Semi-Bantu are more susceptible compared to the Bantu and Foulbe. Several studies have demonstrated differences in susceptibility to malaria, with the Fulani of West Africa having a lower incidence of malaria than other sympatric groups [33] and the Fulani in Burkina Faso being less parasitized than the sympatric Mossi and Rimaibé groups [34]. This is also true for the Malian Fulani and their sympatric neighbours, the Dogon [35]. Various markers confirm that the Fulani are genetically distinct from other African tribes [36]. Such genetic differences may be less apparent in the Littoral and South West regions due to extensive ethnic mixing. However, previous results have also shown that this relative resistance to malaria in the Fulani, as compared to other sympatric tribes, appears to be pathogen related and not due to a general hyper-reactive immune system [37].

The overall case fatality in this study was 4.8%; this was similar by gender (3.3% *vs.* 3.7%), age groups (3.3% *vs.* 3.9%), ethnicity and location. This observation is higher than previous reports in Africa [7,13,14] but may even be an underestimation, as some children might have died at home during the period since a majority of cases of untreated severe and complicated malaria are fatal. The independent prognostic indicator was cerebral malaria confirming the fact that in severe malaria, neurological involvement is the factor that is most associated with poor outcome [7,11,13]. Severe anaemia, though the most frequent presenting feature, was a poor predictor of death [38], perhaps because most severe malaria anaemia children received timely blood transfusion. Despite this, severe anaemia will be an important vaccine trial endpoint because of its frequency, especially in infants and because of the ease with which it can be measured with certainty in field situations.

Even though mortality impact is the most important public health measure of any vaccine efficacy, it represents a smaller proportion of all disease burdens and will, thus, require large sample size in future trials [7]. Again the comparative ease of using the established case definitions of severe malaria [25] and the fact that most children diagnosed as having severe malaria have worse prognoses than those with mild disease makes severe malaria a better trial endpoint. Well-defined severe malaria, therefore, bridges the gap of being common enough for a reduction to be measurable and as important as possible to predict malaria-associated mortality in endemic sites [28].

A significant seasonal variation of the prevalence of severe malaria was recorded over the study period (Figure 3). The highest prevalence occurred in the months of April to October (during the rainy season). The observed pattern points to the fact that increase in vector breeding following the rainy season is responsible for the upsurge in the malarial cases and supports an earlier transmission study [20]. In malaria endemic areas, majority of malaria death and morbidity occur during the peak transmission seasons. As such, the intermittent preventive treatment approaches to malaria control during this intense period could impact on reducing disease burden. Such preventive measures could reduce the risk of severe malaria and subsequently minimize its effect as a vaccine trial endpoint.

## Conclusion

Results from this study suggest that significant hospital admissions and numbers of severe malaria cases exist in the Centre, Littoral and South West regions of Cameroon. In addition, severe malaria in these areas occurs frequently in the first 24 months of life, the predominant feature being severe anaemia while cerebral malaria and hyperpyrexia were the independent prognostic indicator of death. Although the variation of parasitaemia with ethnicity in the Centre region seems to suggest that Semi-Bantu are more susceptible compared to the Bantu and Foulbe, the differences observed may be pathogen related and not due to a general hyper-reactive immune system as previously reported. Such genetic differences may be less apparent in the Littoral and South West due to extensive ethnic mixing.

## Competing interests

The authors declare that they have no competing interests; but they had financial support from MalariaGEN and MIMPAC for the submitted work; no financial relationships with any organizations that might have an interest in the submitted work; no other relationships or activities that could appear to have influenced the submitted work.

## Authors’ contributions

EAA initiated the collaborative project, designed data collection tools, monitored data collection for the surveys, drafted and revised the paper. TOA implemented the surveys, wrote the statistical analysis plan, cleaned and analysed the data, and drafted and revised the paper. RNM implemented the surveys and revised the paper. ANN implemented the surveys and revised the paper. JKA-K implemented the surveys and revised the paper. CNY implemented the surveys and revised the paper. All authors have read and approved the final manuscript.
